# An integrative review of the use of the concept of reassurance in clinical practice

**DOI:** 10.1002/nop2.1102

**Published:** 2022-03-11

**Authors:** Samuel Akyirem, Yakubu Salifu, Jonathan Bayuo, Precious Adade Duodu, Irene Fosuhemaa Bossman, Mary Abboah‐Offei

**Affiliations:** ^1^ Yale School of Nursing Yale University New Haven Connecticut USA; ^2^ Division of Health Research Faculty of Health and Medicine Lancaster University Lancaster UK; ^3^ School of Nursing The Hong Kong Polytechnic University Kowloon Hong Kong; ^4^ Department of Nursing and Midwifery School of Human and Health Sciences University of Huddersfield Huddersfield UK; ^5^ School of Health and Life Sciences Glasgow Caledonian University Glasgow UK; ^6^ School of Health and Life Sciences University of the West of Scotland Scotland UK

**Keywords:** compassion, conceptual framework, emotional care, integrative review, nursing practice, reassurance, therapeutic relationship

## Abstract

**Aim:**

To synthesize evidence on the concept of reassurance in nursing practice.

**Design:**

Integrative review.

**Review Method:**

PubMed, OVID MEDLINE, CINAHL and PsycINFO were searched from their inception to the 30 May 2020. The search results were screened. We assessed the quality of primary studies using the Mixed Method Appraisal Tool. Included studies were analysed using narrative synthesis. The review protocol was pre‐registered (PROSPERO‐CRD42020186962).

**Results:**

Thirty‐two papers out of the 2,771 search results met our inclusion criteria. The synthesis of evidence generated three intricate themes, namely “antecedents of reassurance,” “defining attributes of reassurance” and “outcomes of reassurance.” Emotional distress was the main antecedent of reassurance. The three sub‐themes identified under defining attributes of reassurance include self‐awareness, emotional connectedness and verbal and non‐verbal techniques. Ultimately, reposing the confidence of patients and their families in healthcare professionals and the care delivery process to enable them to overcome their challenges constitutes the outcomes of reassurance.

## 1 INTRODUCTION

The emotional challenges experienced by various patient groups in the clinical setting are well‐documented (Rückholdt et al., 2017). These challenges often range from uncertainty about the course of disease progression, the potential success or failure of treatment modalities, and more generally, the fear of the unknown (Carleton, 2016). Hospitalization often places patients in new and unfamiliar environment, and this serves as a source of anxiety and psychological unrest for patients and their family. Moreover, evidence suggests that patients with chronic pain (Kohrt et al., 2018), long‐term conditions (Holmes & Deb, 2003) and those nearing the end‐of‐life experience hopelessness and loss of confidence in themselves and their healthcare providers (Virdun et al., 2017).

The prevalence of such emotional issues among the various patient groups requires healthcare providers to make psychological care a central part of their daily care routines. One of such psychological care is “reassurance.” Reassurance is vital to a wide‐range of patient groups including those with long‐term medical conditions and those that require palliative care (Sinclair et al., 2017) as well as users of emergency ambulance services (Togher et al., 2015), and patients with non‐specific conditions (Traeger et al., 2017).

Although reassurance as a psychological intervention is widely used in clinical practice, there are some inconsistencies in the characterization of the intervention.

### 1.1 Background

One core duty of the nurse is to provide comfort and allay fears and anxieties of patients and families through therapeutic communication (Pincus et al., 2013). This therapeutic nurse‐patient communication often constitutes reassurance. Reassurance in nursing may refer to the totality of non‐specific actions that are carried out by the nurse and geared towards restoring confidence and hope and reducing uncertainty in patients. It is also said to be the removal of fears and concerns about illness, and may refer to the behaviour of the caregiver or the response of the patient. This nursing intervention is pivotal in carrying out compassionate nursing care (Clarke, 2014).

Nurses provide more hands‐on care and spend the most time with patients than other health professionals do. Westbrook et al. (2011) indicate that the time spent for hands‐on care to patients constitutes more than three‐quarters of nurses’ time. Majority of this time is spent communicating with patients (Yen et al., 2018). This constant and preponderant nurse–patient interaction presents a unique opportunity for nurses to be at the forefront of identifying and helping distressed patients and relatives. It is thus not surprising that nursing documentation is dominated by at least one variant of the statement: “patient was reassured.”

However, despite its widespread acceptance and usage in the nursing profession, reassurance remains a poorly defined term (Rolfe & Burton, 2013), and this was noted decades ago (Teasdale, 1989). Its meaning could range from a reassuring presence of health professionals (Lucas et al., 2008; Traeger et al., 2017) to disclosing information that forecasts positive outcomes (Teasdale, 1989). Another query is whether the form and scope of reassurance changes within a particular framework, such as primary care and clinical care, acute and long‐term settings, as well as the end‐of‐life setting. Some questions remain unanswered about reassurance including (a) what exactly do nurses do to reassure patients (how to reassure), and (b) what nursing actions could patients consider reassuring.

Although a reassurance guide for patients with non‐specific disease exists (Traeger et al., 2017), there has not been a systematic review that comprehensively addresses how nurses reassure patients and what nursing actions and attributes are considered reassuring by patients.

Therefore, this review aimed to explore the state of the evidence regarding the meaning and usage of reassurance in nursing practice and possibly arrive at uniformity in the use of reassurance. We also developed a tentative conceptual framework for reassurance as a nursing intervention. We believe that these would be useful for future nursing research and competence training in nursing education.

## 2 THE REVIEW

### 2.1 Aims

This integrative review attempted to answer the following 3 questions:

1. What is the concept of reassurance as used in clinical practice by nurses?
2. How do nurses reassure patients?
3. What are the outcomes of reassurance in nursing care?

### 2.2 Design

We conducted an integrative review of the evidence on the concept and use of reassurance in nursing practice. For this study, nurses refer to both qualified nurses and student nurses. We followed Whittemore and Knafl’s (2005) updated integrative review methodology, which involves problem identification, literature search, data evaluation, data analysis and presentation. This review is reported in line with the Preferred Reporting Items for Systematic Reviews and Meta‐Analyses (PRISMA) (Moher et al., 2009). The study's protocol was registered on the PROSPERO International prospective register for systematic reviews (CRD42020186962).

### 2.3 Search methods

An electronic search was planned and executed on 30 May 2020 on PubMed, OVID MEDLINE, CINAHL and PsycINFO. PsycINFO helped to cover the psychological nature of the concept under review, CINAHL provided coverage on nursing‐related studies, MEDLINE and PubMed provided wider access to the medical literature. The search was conducted using variants of the search terms: “reassurance” and “nurse.” Both index terms and free texts were incorporated into the search strategy to make our search as sensitive as possible. We limited the search to journal articles and studies with human subjects. No date limit was applied to the search.

The search results were imported into Covidence, a systematic review management software. Subsequently, two reviewers independently conducted title and abstract screening; a third reviewer was consulted where there were disagreements. The use of the three reviewers ensured objectivity in the selection and synthesis of the evidence. The eligibility criteria used were: (a) studies that described reassurance, (b) reassurance delivered by a nurse, (c) patients/relatives as recipients of the reassurance, (d) articles published in English and (e) studies of all designs. We excluded studies that (a) focused on the use of reassurance by other health professionals, and (b) dissertations, abstracts, conference articles and journal articles with no available full text. Full texts of tentatively eligible studies were further assessed to determine whether they fully met the inclusion and exclusion criteria.

### 2.4 Quality appraisal

Since there is no standardized framework for assessing the quality of reflective essays, editorials and opinion pieces, we only evaluated the quality of primary studies. The methodological quality of the included primary or empirical studies was assessed using the 5‐point Mixed Methods Appraisal Tool (MMAT) version 2018 (Hong et al., 2018). The MMAT has proven useful in the critique of qualitative, randomized controlled trials, quantitative non‐randomized, quantitative descriptive and mixed‐method reviews. The tool assesses the appropriateness of a study's aim, study design, participant recruitment, data collection, data analysis, presentation of findings, authors’ discussions and conclusions (Hong et al., 2018). Studies were, however, not excluded based on quality as typical of integrative reviews (Whittemore & Knafl, 2005).

### 2.5 Data abstraction and synthesis

The synthesis of relevant studies was guided by Whittemore and Knafl's five‐pronged approach to data analysis in integrative review: data reduction, data display, data comparison, verification and conclusion. In *data reduction*, included studies were divided into two subgroups: empirical and non‐empirical studies. For empirical studies, we extracted data on the names of the authors, country and study objectives. We also carefully summarized the relevant findings of each empirical study. For the other study types (including reflective essays), we summarized the main ideas or themes highlighted by the papers. The reduced data were then put in a table or data matrix during the *data display* stage. This allowed for patterns across the data to emerge. In *data comparison,* two reviewers inductively coded the data. The codes were then iteratively compared across the studies and categorized into themes. This was done while being mindful of the various patterns and contrasting ideas that are evident in the data. The generated themes were then compared to the reduced data to ensure fidelity to the core meaning of our original data. Conclusions were then drawn about the themes that appropriately and wholly answered our research questions. These last bits of the data analysis process constitute *verification and conclusion drawing*.

## 3 RESULTS

### 3.1 Search outcomes

**FIGURE 1:**
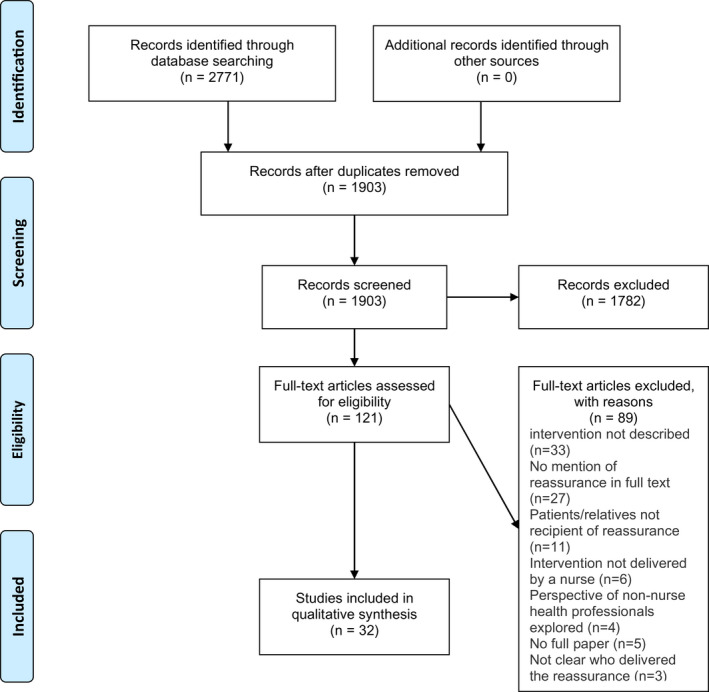
PRISMA flow chart (Moher et al., 2009)

Overall, the electronic search produced *N* = 2,771 results. After removal of duplicates, *N* = 1,903 records remained for the title and abstract screening. *N* = 1,782 articles were removed after the title and abstract screenings and *N* = 121 records were subjected to full‐text review. Out of these, *N* = 87 studies were excluded with reasons depicted in the PRISMA chart (Figure 1), and *N* = 32 studies that met the inclusion criteria were included in the final analysis.

### 3.2 Characteristics of included studies

The details of the included studies are shown in Tables 1 and 2. The review included empirical (*N* = 19) and non‐empirical studies (*N* = 13). The empirical studies were of qualitative (*N* = 14), quantitative (*N* = 3) and mixed‐method (*N* = 2) designs. The empirical studies were conducted in the United Kingdom (*N* = 6), North America (*N* = 6), Australia (*N* = 4) and Sweden (*N* = 2). One study, however, was not clear about the country of origin. The primary studies looked at reassurance from the perspectives of nurses, nursing students, patients and family members. The empirical studies were published between 1976–2019, with most studies being published more than a decade ago (*N* = 12). The sample size used across studies varied considerably (ranged from 1–431).

**TABLE 1:** Summary of evidence (primary studies)

First author (year), country	Study design, aim	Sample characteristics	Findings related to reassurance	Sample codes	Sub‐themes	Themes
Beaver (2005), UK	Mixed‐method study To evaluate the nature and content of patient –HCP interaction in a breast cancer follow‐up intervention	106 women Mean age = 64 years	Friendliness and helpfulness of nursing staff are positive factors associated with reassurance. Women felt reassured when they were physically examined and given confirmation that they were ok	Being nice, physical examination may be reassuring	Nonverbal form of reassurance	Defining attribute of reassurance
Meyer et al. (2019), USA	Qualitative study To investigate clinician’s perspective on communicating diagnostic uncertainty to parents	*N* = 20 clinicians (2 nurses),13 females	Reassurance requires honesty from the nurse. Honesty however does not mean pessimism. The nurse should rather focus on what can be done to address any current concerns the patient may have. “even if we don’t know what’s going on, we usually know what to do, and so I tell them that although I don’t know what’s going on, we do know what to do to make your child better.”	Providing honest information	Verbal forms of reassurance	Defining attribute of reassurance
Karlsson (2012), Sweden	Hermeneutic qualitative study To observe, interpret and describe nurses’ communication with conscious patients in an intensive care unit	6 women, 13 men Mean age = 59 years	The nurse ensures reassurance by demonstrating that she knows what she’s doing and by conducting her nursing actions calmly and methodically. Talking quietly to patients is equated to reassurance	Display of competence is reassuring, being calm	Professional competence, non‐verbal form of reassurance	Defining attribute of reassurance
Al‐Mutair (2014), Australia	A descriptive exploratory qualitative design To identify the perceived needs of Saudi families of patients in Intensive Care in relation to their culture and religion	*N* = 12, 10 males; mean age = 44.25	The provision of honest and ample information by the HCP was seen by relatives as reassuring. The use of optimistic/encouraging words, nonverbal cues (eye contact, varied facial expression, smiling face), by ICU HCP were seen as reassuring by family members in the ICU “As a second patient (he called the family member the second patient) we need care, hope, optimism, use of encouraging words and we need the caregivers to take into account the humanitarian aspect … sometimes the information made us tense and prevents us from sleep … you can give honest information with a bit of optimism. Don’t lie or deceive, we all know that all ICU patients are critical but how to offer suitable words? I think they [healthcare providers] need courses to do this” Patients' relatives also found it reassuring when they are involved in the care of their sick patient. “If I’m given the choice, I would sit in front of him [father] all the time, feed him, take care of him, talk to him, read Qur’an for him, I would stay beside him even if he is sleeping; at least this will reassure me and reduces my anxiety but unfortunately, this is not allowed”	Adequate and honest communication (focus on relatives), use of verbal and non‐verbal cues, instilling a sense of hope, use of encouraging words, caregiver inclusiveness in the reassurance process, family reactions to clinical information, instil hope but avoid deception, improving staff communication	Verbal and non‐verbal forms of reassurance, instilling hope, family‐centredness	Defining attribute of reassurance, consequence/outcome of reassurance
Hicks et al. (2014), UK	A two‐arm pilot randomized controlled trial This pilot aimed to test study procedures and inform sample size for a future multi‐centre trial and to gain initial estimates of the effectiveness of the discussion intervention	120 patients with chest pain About 40% of patients attending the clinic took part in the study	A 5‐item patient‐reported scale was used to measure the level of reassurance at month 1 and 6 (higher score indicating higher levels of reassurance). Validity of the questionnaire (Cronbach’s alpha = 0.68) Although there was no significant difference between those who discussed with a nurse and those who received pamphlets alone, there was evidence of high levels of reassurance reported. That suggests that reassurance can be in the form of discussion and giving information (pamphlet). So reassurance could be delivered in a form that meets the patient’s needs. However, this (pamphlet method) may not be applicable in settings where the patients cannot read, or resources cannot support the same.	A tool for measuring reassurance?, means of delivering reassurance as an intervention, patient‐centredness, method of delivery based on settings	The verbal form of reassurance, self‐awareness	Defining attribute of reassurance
Cossette et al. (2002), Canada	Quantitative study (secondary analysis) This paper examines the types of nursing approaches associated with reductions in psychological distress in postmyocardial infarction patients	Involved 431 (36.2% women) of the 692 patients in the M‐HART treatment group	Three types or main approaches found to be emotionally supportive and led to a reduction in distress (NB only the part needed for this work was retrieved). Reassurance/encouragement, listening and the provision of advice. Authors contend that the approaches may work differently for different people and may vary based on gender. Sometimes, listening to a patient is enough reassurance	Encouragement, listening, counsel, patient‐centredness, the method used depends on the setting	Verbal/Nonverbal form of reassurance, self‐awareness	Defining attribute of reassurance
Gustafsson (2018), Sweden	Descriptive interpretive design To explore the need for patients to feel reassured when receiving telephone nursing for minor illnesses?	*N* = 11 patients	Patients feel reassured when they see the nurse as another human being who understands their situation and sympathizes with them. This is often achieved by the nurse sharing their personal experiences with patients and “coming to the level of patients.” Participants felt reassured when the nurse was professional, calm and factual. The nurse being alert and asking the right questions was seen as reassuring. Patients get anxious when the cause of the symptoms is unknown. Patients feel reassured when a competent nurse provides a clear explanation of the symptoms to allow them to have a good picture of their risk level and the actions they need to take. Provision of clear advice on what the patient must do was considered a reassuring activity by patients Patients feel reassured when their concerns are not taken as trivial. It was reassuring to patients when the nurse deals with any “minor” complaint with all seriousness	Demonstrating empathy, sharing of lived experiences and connecting with the patient, demonstrating competency, being authentically present, cause of anxiety, providing clear explanations, handling patient’s concerns	Emotional/ situational connectedness, professional competence, verbal/nonverbal form of reassurance, emotional triggers	Defining attribute of reassurance, antecedent of reassurance
Boyd (1989), USA	Qualitative study design To explore how nurses offer reassurance to patients	15 female registered nurses	Reassurance is mostly needed by patients when they start going through uncertainty about their health or treatment/intervention outcomes. Most nurses initiate the process of reassurance upon seeing signs of distress in patients (crying, restlessness and asking plenty of questions. Other nurses are a little more “presumptuous.” Such nurses analyse the facts of the client's situation and assume that the client must be going through so much distress that requires reassurance. Though this may indicate that the nurse is emotionally availing herself to assist the patient, the nurse also risks projecting her feelings wrongly onto the patient Nurses reassure patients by giving them factual and accurate theoretical information. This included providing results of lab tests, explaining procedures and using theories to validate patients' experiences as normal. Other ways include providing therapeutic touches (and other physical comforts), crying with clients, carrying out tasks confidently and calmly, staying with clients and listening to them	Patient’s uncertainty as a starting point of reassurance, emotional distress, identification and analysis of client’s distress, therapeutic use of the nurse’s “self,” providing accurate information to patients, normalizing the patient’s status	Emotional triggers, self‐awareness, verbal/nonverbal form of reassurance, emotional balance/ acceptance	The antecedent of reassurance, and defining attribute of reassurance
Brockway Plummer & Lowe (1976), USA	Quantitative study To compare the effects of 2 types of nursing reassurances (superficial vs knowledgeable) on patient anxiety, as measured vocally by the Psychological Stress Evaluator	23 patients (pregnant women) Each patient served as their control (vocal stress measurement)	14 received “knowledgeable” reassurance (R2) 9 received “superficial” reassurance (R1). 3 persons who received R2 showed decreased vocal stress level	Reducing stress	Goal of reassurance	Outcome/consequences of reassurance
Usher and Monkley (2001), Australia	Qualitative study To explore nurses’ perspectives of effective communication in the ICU	10 Registered Nurses interviewed	The physical presence of ICU nurses around patients is seen as reassuring. Reassurance may also require non‐verbal cues such as touch, massage, holding hands. Patients feel reassured when they see the nurse as another human being with emotions just like them. Nurses can reassure patients by disclosing their own emotions to patients. Reassurance requires that the nurse forms a trusting relationship with patients. The nurse achieves this by being honest with the patient and upholding patients’ dignity and confidentiality. The provision of accurate information was also seen as reassuring. Such information may encompass details of treatment, the nature of the patient’s condition and ensuring that the patient is well oriented to his environment	Being present, non‐verbal techniques, connecting with the patient, sharing personal lived experiences, trust, honesty, keeping patients informed	Non‐verbal/verbal form of reassurance, emotional connectedness, maintaining personhood/identity	Defining attribute of reassurance
Fareed (1996), UK	A qualitative study using a phenomenological approach The aims of this study were as follows to identify which nursing interventions were found to be reassuring by the patients, to identify the outcomes of reassurance on the patients' state of well‐being and to be able to answer the question of how can nurses know that patients are reassured”	A medical and a surgical ward were used to recruit: a wide spread of age differences, and a mixed group of informants with heart and lung conditions, rheumatology, gastroenterology and vascular disorders (in acute mixed medical unit); and gastro‐intestinal disorders, urology and vascular surgery (in the acute mixed surgical ward of a local general hospital unit)	Themes that emerged included receiving information and knowledge of facts, use of appropriate interpersonal and communication skills, nurses' presence, “being cared for,” a trusting relationship, an assertion of optimism and a perceived therapeutic environment. Patients seem to feel the therapeutic effect of reassurance where the environment in which they are nursed is seen to be informal, unthreatening and caring. These are characteristics of a ward, where the staff are friendly, kind, pleasant and where patients are encouraged to express their feelings. The effects of reassurance are perceived as consisting of two components—external and internal factors. The external factor refers to the perception of things outside of the person, whereas the internal factor refers to temporality. Since the loss of control is evident during hospitalization, gaining control over the situation one is in, is a form of reassurance The presence of the nurse was highlighted as an important factor in reassurance. The nurses did not physically have to be next to the patient The knowledge that they were accessible was enough to convey a sense of security	Keeping patients informed, interpersonal skills, being present, optimism, therapeutic use of the environment, being nice, verbalizing emotions, hospitalization, reassurance means gaining control, sense of security	Verbal/Nonverbal form of reassurance, connectedness, instilling hope, ward environment, emotional triggers, emotional balance/ stability, emotional connectedness, sense of security	Defining attribute of reassurance, consequence of reassurance, antecedent of reassurance
Gibb & O'Brien (1990), Australia	An ethnographic study What inferences can be made about the way registered nurses relate to elderly residents in their care, from conversational interaction associated with nursing care activity?	Ten registered nurses, who were employed at two nursing homes in the Geelong area took part voluntarily in the study Time in employment vaned from between 1–6 years	From the results, it is clear that speech style vanes partly as a function of the physical activity engaged in by the nurse, and that one of its major functions is in the efficient execution of that activity as a cooperative endeavour. Obvious samples were the predominance of instruction during toileting, where the active participation of the resident was mandatory, as well as the predominance of a personal probing questioning style during the execution of a clinical procedure such as an examination. Concerning the various speech styles related to various aspects of morning‐care activities, it may be considered that each gives a different view of a general kind of relationship that nurses have with residents of a nursing home, developed within this cultural framework. In general terms, that relationship may be characterized as one of support, encouragement, directiveness and the communication of personal respect for the client without extensive personal interest on the part of the nurse	Communication, Competency	The verbal form of reassurance, forms of reassurance (professional competence)	Defining attribute of reassurance
Wocial et al. (2014), USA	Qualitative design (focus group discussions; the deductive approach used to generate data) A total of 39 nurse focus groups were conducted with 363 participants, and an additional 63 direct care nurses completed online surveys To explore the meaning of the phrase “image of the nurse” in the context of the desired brand experience of assurance	Nurses working and employed in the inpatient and ambulatory care areas of any Indiana University Health facility Nurses in traditional roles (e.g. direct care providers, educators, managers, directors, care coordinators and clinical nurse specialists) were eligible to participate, as were nursing students	The theme of assurance is complex. The most significant and consistent meaning found in the data was that rather than a specific colour, it is a nurses’ overall appearance, being clean, well‐groomed and wearing a modest well‐fitting uniform that establishes the tangible elements of the brand of assurance Interpersonal skills can reinforce the skills and behaviours of nurses and communicate assurance Nurse behaviours that reassure patients include being present with patients, helping patients know what to expect and demonstrating a consistent team approach Nurses are expected to be “genuine (representing approachable) and advocate (representing professional). Being genuine included many different things such as being respectful, attentive and caring. Being an advocate included being informative, reliable and being collaborative with other members of the healthcare team.” When asked “What would assure you that your loved one was being cared for by an ideal nurse?” beyond behaviours, participants identified characteristics of a nurse. Important traits of a nurse included being compassionate, approachable, attentive and caring. Having good manners and being gracious were other ways participants described nurses who assured patients	The complexity of the concept, nurse’s appearance conveys reassurance, interpersonal skills, being present, keeping the patient informed, showing genuine interest, compassion, approachable, caring	Nature of reassurance, physical appearance, emotional/ situational connectedness, verbal form of reassurance, emotional connectedness, nurse’s attributes	Defining attribute of reassurance
Hermann et al. (2019), USA	Qualitative descriptive design To describe patients’ experiences of their communication with nurses and providers in the emergency department and fast track and identify potential best practices	A stratified sampling strategy was applied to enrol 2–4 women and 2–4 men from each of 4 age brackets (18–29, 30–44, 45–65, 66) from both the emergency department and fast track. A total of 30 participants completed the interviews	Based on the study findings, “reassurance” was defined as recognizing participant’s anxiety, fear, or lack of ability to carry out the treatment plan and addressing concerns by responding to their emotional needs. Participants expressed appreciation when nurses and providers conveyed this reassurance by acknowledging their fears and telling them, “Don’t be afraid” or “You are going to be OK.” One participant reiterated “They were in command of their discipline in terms of knowledge and understanding on how to treat people.”	Definition of reassurance, acknowledging patients’ fears is reassuring, reassuring words, demonstrating competence	Emotional triggers, verbal forms of reassurance, forms of reassurance (professional competence)	The antecedent of reassurance, and defining attribute of reassurance
Jay (1996), UK	Qualitative design To explore and describe issues in relation to nursing care that are important to trauma patients in A & E	Five seriously injured patients were interviewed several days after admission. This sample size was increased to include two members of staff	Central to the delivery of emergency care is the individual’s transition from their normal independent existence through pre‐hospital trauma and into the isolating experience of fear, dependence and the resuscitation room. Different methods of coping were required. Methods such as touch, company and information became paramount as did the need to trust the people seen to be in control	Awareness of the patient/ family transitions during illness, nonverbal technique, communication and trust	Family‐centredness, self‐awareness, verbal/non‐verbal form of reassurance	Defining attribute of reassurance
Jones et al. (2007), Australia	Qualitative study To examine mothers’ and fathers’ perceptions of effective and ineffective communication by nurses in the neonatal intensive care unit (NICU) environment	20 mothers and 13 fathers participated in the semi‐structured interviews	The findings suggest that reassurance can be a mode by which nurses within the NICU environment can most effectively communicate with parents, in addition to listening and asking for input/ suggestions	Active communication	The verbal form of reassurance	Defining attribute of reassurance
Teasdale and Kent (1995), UK	Critical incident technique/qualitative study To provide empirical data on the use of deception in clinical settings	251 nursing staff participated in the critical incident review. 20 nurses participated in the follow‐up interviews	Reassurance reflects a nursing action that includes emotional support and distraction	Nursing action, Distraction	Non‐verbal form of reassurance	Defining attribute of reassurance
Chauhan (2000), [Country not stated]	Qualitative design (role‐play analysis) To examine how nurses break bad news to patients and give information via reassurance	19 MSc Nursing students, 7 minority	Distressed patients may trigger emotional upset in the nurse (perhaps through the nurse showing extreme sympathy). In reassuring patients, nurses must become self‐aware and be truthful to their humanness, vulnerabilities and unresolved emotions and make efforts to distinguish their own needs from that of the patient. Some nurses in an attempt to reassure patients may end up reassuring themselves. The portrayal of “extreme” sympathy could be a barrier to reassurance. Nurses sometimes give false assurances to distressed patients in an attempt to give them hope. It is important that the nurse first identify what patients are worried about and why they are concerned before reassurance begins. Non‐verbal techniques such as therapeutic touch (shoulder and arm), prolonged eye contact proximity, active listening, the calm voice may be useful. Cultural differences in the interpretation of touch exist and so the nurse must use this method wisely	Distressed patients make nurses emotionally uncomfortable, being truthful to oneself, nurses should not project their needs, nurses as beneficiaries of their reassurance, barrier to reassurance, lying to the patient, restoring hope, identifying the source of emotional distress, non‐verbal forms of reassurance, being culturally sensitive	Emotional triggers, self‐awareness, emotional balance/ self‐awareness, forms of reassurance (ineffective), emotional triggers, non‐verbal form of reassurance	The antecedent of reassurance, defining attribute of reassurance
Barr (1992), UK	Case study (Qualitative) To outline the nursing management of a patient with breathing difficulties	A 75‐year‐old woman with congestive heart failure and COPD	The patient found it reassuring when the nurse held her hands The nurse reassured the patient by “normalizing” and accepting the patient’s emotions and reactions (e.g. making the patient feel that she is not being a nuisance by repeatedly requesting for bedpan) The nurse reassured the patient by ensuring that the patient's pre‐illness identity, dignity and self‐esteem remained intact. These were expressed in both deeds and words The nurse also provided words of encouragement to patients. (such as “she will recover,” “she will wake up if she goes to sleep”)	Non‐verbal forms of reassurance, recognizing and accepting patients’ feelings as an expected response, the goal of reassurance, reassurance goes beyond what you say, encouragement	Nonverbal form of reassurance, situational awareness/acceptance, maintaining personhood / Identity**, n**on‐verbal form of reassurance	Defining attribute of reassurance, outcome of reassurance

**TABLE 2:** Summary of evidence (nonempirical studies)

Citation	Reduced data (written summary)	Codes	Sub‐themes	Themes
Gregg (1955)	The purpose of reassurance is to help the patient restore confidence in himself and his ability to solve his problems. Patients feel reassured when: indecisive feelings disappear, someone listens to their problems, they feel respected, accurate information is provided, the patient observes technical competence in the nurse The nurse's role in reassurance involves identifying signs of distress in patients (overt signs such as facial expressions, crying and covert signs such as the type of questions patient asks), not making light of patients problems (by avoiding the usage of phrases such as “it is not that bad,” or “you will be fine” ‐ “reassurance noises,” asking probing questions while showing genuine and sincere interest in helping the patient, maintaining a non‐judgemental attitude) In the end, the patients must be empowered to find solutions to their problems. Diversionary measures, therefore, may be a less useful strategy	Reassurance definition, the outcome of reassurance, listening, respect, keeping patient informed, demonstration of competence, identify signs of distress, reassuring noises, showing genuine interest non‐judgmental, acceptance, empowering patients, diversion	Forms of reassurance, emotional distress, emotional connectedness, acceptance	Defining attribute of reassurance, consequence/ outcome of reassurance
Blackhall (2011)	Reassurance involves the provision of information to patients. The VERA framework as a guideline for compassionate communication could also provide a skeletal framework for reassurance as a nursing intervention. The first step is to validate or accept patients' perception of their problem in a non‐judgmental way and the patient is encouraged to verbalize their problems. The next step is to establish an empathetic connection with the patient. Reassuring words are then provided that everything will be all right. Lastly, an activity is constructed to integrate patients’ emotions rather than invalidating them	Keeping the patient informed, non‐judgmental, acceptance, encourage patient to voice out, empathy, everything will be all right	Forms of reassurance	Defining attribute of reassurance
Monk (2019)	Nurses reassure by being present, talking to patients, allowing questions to be asked and providing therapeutic touches Reassurance involves making patients feel safe and understood	Being present verbal/non‐verbal actions	Forms of reassurance	Defining attribute of reassurance
French (1979), UK	Reassurance may be carried out by any individual in interpersonal interaction, although it is suggested that the nurse can develop some expertise by experience and training The nurse should be able to recognize verbal and non‐verbal behaviour in another person which indicates that the individual may be apprehensive or anxious including: knowledge of the functions of patients' questions and remarks. the observable physiological manifestations of the anxiety response; social reactions to anxiety; psychological reactions to anxiety. The nurse should list and anticipate those situations in which patients commonly lose confidence. The nurse should be able to describe and carry out a repertoire of behaviours that she may use to attempt to restore the person's confidence. The nurse should be able to identify when these behaviours have been successful, and state what she will do when they are not successful. Behaviours twitch may need to be adopted to achieve reassurance include: Explanation—It involves the provision of information about anxiety‐provoking situations and the future. Familiarizing an unfamiliar situation—the escorted tour of the ward which some nurses allow the patient on admission. Introducing a familiar element to unfamiliar situations—admitting the mother with her child, and allowing the patient's possessions, clothing, toys, photographs, etc., around him in hospital. Touch—human contact is a form of nonverbal communication that can provide comfort. Proximity (physical presence of the nurse)—the mere presence of a fellow human being can provide reassurance when loneliness may cause more apprehension Conveying emotional stability in the nurse's manner using non‐verbal communication—If the patient identifies anxiety or apprehension in the nurse it can confirm his fears or lead him to suspect actual danger or problems. Counselling and helping patients to use their skills to overcome fears—this can engender the feeling of having control over the situation. Clarification of facts—This is similar to explanation, but the emphasis is on placing the patient's knowledge of his disease or prognosis in the correct perspective. Verbalization and ventilation of fears by the patient—a verbal expression of doubts and fears by the patient is important in reassurance. Diversional techniques—a conversation with others, group activities, recreational activities and occupational therapy. The nurse can provide these situations. Portraying the expected role—if an individual nurse's appearance or behaviour does not fit the patient's expectations it will cause some apprehension Knowledge and competence—Inefficiency, incompetence and clumsiness rarely inspire confidence. The concept of reassurance should not be taken for granted. It should be adopted as a nursing skill and not be regarded as a vague phenomenon that is achieved in some magical way. All nurses should attempt to realize the activities which they may perform to attempt to reassure people (patients, relatives and colleagues alike)	Context of reassurance, competency, recognizing patient's cues (need/context of reassurance), nursing actions to attain reassurance, environment, physical presence, emotional stability, empowerment/gaining control, keeping patients informed, diversion, competence	Emotional distress, forms of reassurance, self‐awareness	Defining attribute of reassurance, antecedent of reassurance
Halm (1992) US	Support and personal needs have been empirically validated as two of the most important family need categories during critical illness. Perhaps overshadowed during the initial critical care phase by a need for relief of initial anxiety and reassurance of quality care and information, support needs emerge as family members recognize the impact of the stressful illness experience on themselves. Critical care nurses can provide social support to family members through family assessment, counselling and support groups. Although not empirically tested, it is generally believed that such support will influence the ability of family members to provide support to the patient and thereby influence a positive recovery from critical illness.	Social support to family and critically ill patients		Antecedent of reassurance
Diggins (2012) USA	Reassurance can be uncomplicated as smiles, simple touch and the presence of a healthcare professional to ease and calm the fears of patients. A key component of the reassurance process also includes clear communication of patients’ condition or results to them. Patient care transcends beyond prescription, treatment or further diagnostic testing. However, the religious faith of individual impacts immensely on the way reassurance is provided It is possible to think “when patients don't need a prescription, treatment, or further diagnostic testing, I sometimes think I have offered them nothing. I didn't “do” anything for them. But then I see the relief on a mother's face after I’ve given her my input.” Reassurance goes beyond prescriptions and diagnostic procedures	Non‐verbal actions, honest communication, religious considerations, reassurance leads to relief, openness, reassurance transcends medical treatment	Forms of reassurance, self‐awareness, nature of reassurance	Defining attribute of reassurance
Easby (2013) UK	One non‐negotiable skill required in the nursing profession is effective communication, which is equally a key fulcrum to the reassurance process. Particularly, patients undergoing surgery or with special needs such as learning disabilities may need such effective communication skills to provide caring and compassionate reassurance. Inexperienced or in‐training healthcare professionals, and even experienced professionals, may at certain times need to overcome their fears and anxieties to reassure patients Similarly, they may be faced with the difficulty of expectations of promises to patients regarding the outcome of a health intervention as part of the reassurance process	Effective communication, overcoming one's fears, patient's expectations	Self‐awareness	Defining attribute of reassurance
Fareed (1994) UK	“The phenomenon of “reassurance” is an attribute of caring that is commonly used in the delivery of nursing care. If caring is considered central to the concept of nursing, a case is made that the therapeutic value of reassurance needs to be analysed.” Reassurance is used as a general term in everyday life. It is central to care, which in turn underlines nursing practice. However, its philosophical underpinnings in nursing care need to be explored. It has been widely reported to contribute to coping mechanisms by patients and therefore there is a need to explore how exactly this happens. It has been described as both therapeutic and non‐therapeutic in literature. Different uses have been described and need to be explored contextually. Optimistic assertions are sometimes equated to reassurance and require in‐depth exploration. “It seems therefore that reassurance is an intimately bound attribute of the caring notion. This is a very important issue since it is claimed that 'caring is the essence of nursing'**”** “It is necessary to examine the effects of reassurance on coping because it seems that assumptions are made that when someone (or a patient) is given reassurance he/she is more able to come to terms (or cope) with whatever was causing the conflict. This assumption is not only weak but also takes a mechanistic (cognitive) view of the person.” The Concise Oxford English Dictionary defines reassurance (as a noun) as “renewed or repeated assurance, renewed or restored confidence,” and (as a verb) “to restore confidence, to remove the fears or doubts of.” **“**Some authors posit that reassurance might be termed “a technique for handling anxiety when it refers to a purposeful, skilled therapeutic move interpersonal relations.” However, others take a contradictory view, labelling reassurance as a “non‐therapeutic technique” The concept of reassurance was analysed using a structured approach, and the authors came out with three uses of the term in healthcare settings: “reassurance as a state of mind,” “reassurance as a purposeful attempt to restore confidence” and “reassurance as an optimistic assertion” “In using the term reassurance to mean 'a purposeful attempt to restore confidence', Some authors took a behavioural approach by elaborating on the activities that the nurse should do to perform the act of reassuring, or what the patient should do to restore assurance These activities include explaining, familiarizing an unfamiliar situation, introducing a familiar element to unfamiliar situations, touch, proximity (physical presence of the nurse), conveying emotional stability in the nurse's manner using non‐verbal communication, counselling and helping the patients to use their skills to overcome fears, clarification of facts, verbalization and ventilation of fears by the patient, and diversional techniques.”	Reassurance is associated with caring	Nature of reassurance	Defining attribute of reassurance
Scott (2006), UK	This was a reflective account of a student nurse's experience of dealing with distress from the death of a patient on the ward. The reflection expressed a positive reassuring experience when one's emotions were recognized with encouraging remarks. “..I feel reassured that it is acceptable to express my emotions, should I need to”	Expressing one's emotions	Acceptance	Defining attribute of reassurance
Teasdale (1995), UK	The study explored the concept of reassurance in healthcare settings by conducting a literature review on the different types of anxiety‐management interventions and classified them under four major strategies: uncertainty reduction, patient control, cognitive re‐framing and using an attachment. The author considered the following strategies more reassuring: using cognitive interventions that allow patients to have positive perceptions about situations that they initially considered as threatening. the use of supportive attachment relationships to make patients feel confident and safe. The study further asserted that reassuring techniques should allow patients to remain passive and reduce their anxiety. Hence, anxiety‐reducing strategies like “patient control” which empowers patients to take action for themselves were not considered as reassuring. This reiterates the idea that “patient autonomy is not always compatible with reassurance.”	Types of reassurance, enabling positive appraisal of a situation (outcome), making patients feel confident and safe (outcome), patient passivity	Nature of reassurance	Defining attribute of reassurance, consequences/outcomes of reassurance
Price (2017), UK	Patients who are scheduled for medical interventions are normally anxious. Nurses are encouraged to attempt reassurance for these patients by sharing detailed information about the planned procedure to relieve their anxiety. This is in line with the NMC Code to “prioritize people” by acknowledging when they are in distress and caring compassionately. Patients especially feel reassured when they receive information and clarity from HCPs who will be involved in the specific intervention.	Identifying distress states and responding compassionately, communication	Emotional triggers, forms of reassurance	The antecedent of reassurance, and defining attribute of reassurance
Davidhizar & Cramer (2002) USA	Client education is reassuring Client education ensures good patient outcome, satisfaction and meeting standards. For client education to be successful self‐assured manner of the communication individualized teaching always giving the rationale for the care self‐take during procedure communicating at the level of the patient note cultural variation medication use “The best thing about the hospitalization was the nurses telling me what they are doing and why they are doing it! It was really reassuring to have the procedures explained and to know what is being done and why.”	Communication/keeping patients informed	Form of reassurance	Defining attribute of reassurance
Teasdale, UK, 1989	Several meanings for reassurance are identified in both nursing literature and practice. Reassurance had three main definitions identified in the Oxford English Dictionary as: (a) Renewed or restored confidence, (b) renewed or repeated assurance and (c) reinsurance. The first two are used in nursing whereas the third form “reinsurance” is not employed in nursing. The author conducted a concept analysis of the term “reassurance” and its occurrences in Nursing times in 1986. Three main usages of the term were identified in clinical settings in addition to the dictionary definitions. These included: Usage 1‐reassurance as a state of mind: as a noun, it explains a state of renewed or restored confidence. An example suggesting that a state of reassurance has been achieved is when a patient states “I was really worried before you told me that, but now I know that I have nothing to fear.” Usage 2‐reassurance as a purposeful attempt to restore confidence: used in a verb form to act purposefully to restore a person to a state of confidence. Usage 3‐reassurance as an optimistic assertion. This is a less common usage than the first two. Unlike usages 1 and 2, which emphasize “states” or “outcomes,” usage 3 stresses an “action.” As a noun, it means a renewed assurance given by one person to another. In practice words such as “don't worry” or “we'll take care of you” may be considered as ways to purposefully attempt restoring confidence to patients The controversy in literature arises from the inability to differentiate between the three usages of reassurance. Some researchers had classified usage 3 as non‐therapeutic. Understanding the concept of reassurance in nursing requires identifying it's specific usage and supporting evidence from published accounts	Restoring confidence, Intentionality	Nature of reassurance	Consequence/ outcome of reassurance, defining attribute of reassurance

The non‐empirical studies included conceptual papers and reflective essays that respectively provided in‐depth analyses of the concept and the accounts of personal experiences on the application of reassurance in nursing care. Across primary and non‐empirical studies, reassurance was discussed in the context of compassionate nursing care and effective patient–nurse communication.

### 3.3 Results of the data evaluation

The Supplemental Tables summarize the results of the quality appraisal of included studies. Overall, the studies had clear research questions and collected appropriate data. For the qualitative studies, the data analysis and interpretation were rooted in the data as evident by the various verbatim quotes used to support the data interpretation. For one qualitative study, it was unclear whether the findings were adequately derived from the data and whether any interpretation could be substantiated by the data (Jay, 1996). The quantitative studies were of sound methodological rigour except for the high risk for non‐response observed in one cross‐sectional study (Cossette et al., 2002). One study, which used a mixed‐method design, did not provide any explicit justification for using both quantitative and qualitative methods (Beaver & Luker, 2005). The mixed‐method studies did not highlight and explain any divergence in quantitative and qualitative data.

### 3.4 Synthesis of evidence on reassurance

Three themes were generated from the synthesis: antecedents, defining attributes and outcomes of reassurance.

#### 3.4.1 Antecedents of reassurance

The theme describes the events that herald and necessitate reassurance. The presence of emotional distress in patients and their families emerged as a significant event that led to reassurance (Boyd & Munhall, 1989; Fareed, 1996; Gustafsson et al., 2018; Halm, 1992). Emotional distress is associated with hospitalization (Fareed, 1996; Halm, 1992), unknown intervention or treatment outcomes (Boyd & Munhall, 1989), unknown symptoms (Gustafsson et al., 2018), uncertainty about one's health (Boyd & Munhall, 1989) and patient's perceived inability to execute a treatment plan (Hermann et al., 2019). Emotional distress can manifest either overtly or covertly, which triggers a commensurate compassionate response from nurses (Halm, 1992; Price, 2017). Overtly, distress can manifest as crying, altered facial expressions (Boyd & Munhall, 1989; Gregg, 1955) and restlessness (Boyd & Munhall, 1989). Covertly, distress manifestations include the patient asking several questions, which may be incongruent with prevailing health needs (Boyd & Munhall, 1989; Gregg, 1955), fear and anxiety (Gustafsson et al., 2018; Hermann et al., 2019) and non‐verbal behaviours of apprehension (French, 1979). The subtleness of these manifestations may suggest that reassurance is an active approach to the identification and resolution of a distressing episode (Boyd & Munhall, 1989; Hermann et al., 2019; Price, 2017).

#### 3.4.2 Defining attributes of reassurance

The theme focuses on the characteristics of the concept. Reassurance is a complex concept characterized by the use of interpersonal skills to intentionally develop and maintain emotional connectedness with the patient and family (Fareed, 1996; Wocial et al., 2014). It requires that the nurse demonstrates self‐awareness, emotional connectedness and apply verbal and non‐verbal techniques to help restore confidence and empower patients as described below:

### 3.5 Self‐awareness

Self‐awareness is regarded as preliminary to the actual reassuring actions undertaken by the nurse. Reassurance requires the nurse to be consciously aware of her emotions and the religious and/or cultural context within which he/she operates. Anxious patients may make nurses apprehensive (Chauhan & Long, 2000). Thus, it is likely that the nurse may be reassuring him/herself in an attempt to reassure the patient (Chauhan & Long, 2000). Also, the nervous nurse is at risk for projecting his/her emotions to the patient (Boyd & Munhall, 1989) as well as triggering an exacerbation of the patient's fears (French, 1979). Therefore, the nurse needs to acknowledge or even overcome his/her emotional state, vulnerabilities or humanness and make attempts to make the patient the centre of his/her reassuring efforts (Chauhan & Long, 2000; Easby, 2013). The nurse, in effect, can provide effective patient‐centred reassurance if he/she remains true to his/her emotions or self. Also, the choice of reassurance technique depends on the culture (Chauhan & Long, 2000), geographical settings (Cossette et al., 2002; Hicks et al., 2014) and religious affiliation of patients (Diggins, 2012). The nurse provides appropriate, acceptable and effective reassurance by demonstrating cultural and religious sensitivity or awareness (Barr, 1992; Chauhan & Long, 2000; Diggins, 2012). For instance, therapeutic touch may not be appropriate in certain cultural contexts especially when delivered by the opposite sex (Chauhan & Long, 2000).

### 3.6 Emotional connectedness

This sub‐theme discusses the need to establish an emotional connection with patients during reassurance. Through this connectedness, nurses avail themselves emotionally to connect with the patient (Boyd & Munhall, 1989; Gregg, 1955; Jay, 1996; Teasdale & Kent, 1995; Wocial et al., 2014) within an enabling environment (Fareed, 1996). Emotional connectedness and compassion facilitate the sharing of each other's lived experiences through which the nurse can recognize and accept the patient's emotions (Barr, 1992; Wocial et al., 2014). The nurse comes to the level of the patient and shares with the patient personal stories that resonate with him/her (Gustafsson et al., 2018; Usher & Monkley, 2001). This strengthens the nurse–patient bond and allows the patient to freely verbalize his/her concerns and develop trust in the nurse. The emotional connection is also facilitated by the nurse showing genuine interest in the patient's concerns, being honest, respectful, caring, empathetic and non‐judgmental (Blackhall et al., 2011; Gregg, 1955; Wocial et al., 2014). These nurse attributes constitute some of the factors that influence the success of reassurance. Aside from the emotional attributes demonstrated by the nurse, physical appearance is also of the essence as it may convey reassurance (Wocial et al., 2014). The relationship that emerges makes the patient and family feel safe (Fareed, 1996), develop trust in the practitioner (Jay, 1996), able to acknowledge their fears or emotional distress (Hermann et al., 2019) and work towards acceptance (Gregg, 1955).

### 3.7 Verbal and non‐verbal forms of reassurance

Another key characteristic that emerged from the data reflects the forms of reassurance that enable the patient and family to regain their emotional balance or stability (Fareed, 1996). The forms were broadly categorized as verbal and non‐verbal. Non‐verbal forms of reassurance included therapeutic touch, maintaining eye contact, active listening, calm voice (Al‐Mutair et al., 2014; Barr, 1992; Boyd & Munhall, 1989; Chauhan & Long, 2000; Cossette et al., 2002; Karlsson et al., 2012); being nice (Beaver & Luker, 2005); being authentically present with the patient and family (Fareed, 1996; Monk, 2019; Usher & Monkley, 2001); and demonstrating respect (Gregg, 1955).

Verbal forms of reassurance noted in the data included offering words of encouragement (Al‐Mutair et al., 2014; Barr, 1992; Hermann et al., 2019), and demonstrating professional competence (Gibb & O’Brien, 1990; Gregg, 1955; Hermann et al., 2019; Karlsson et al., 2012). Other forms of reassurance were communicating adequate, clear, honest and accurate feedback (Al‐Mutair et al., 2014; Boyd & Munhall, 1989; Cossette et al., 2002; Gibb & O’Brien, 1990; Gustafsson et al., 2018; Jones et al., 2007; Usher & Monkley, 2001); and keeping the patient and family informed and encouraging them to verbalize their concerns (Blackhall et al., 2011; Fareed, 1996; Wocial et al., 2014). This encouragement should, however, be devoid of deception, as such practices amount to false reassurance which has been shown to be ineffective, and in some cases detrimental to patients’ well‐being (Chauhan & Long, 2000). Gregg (1955) also noted that the generic and mundane use of certain verbal responses such as “you will be fine” and “it is not that bad” may be regarded as “reassurance noises.” The nurse should use such words cautiously (especially before any proper assessment of patient's complaint is done) as it may suggest to patients that their problems are being belittled.

#### 3.7.1 Outcomes of reassurance

This theme highlights the outcomes or consequences of reassurance in patients. A common expected outcome of reassurance is the restoration of patients’ confidence in their ability to find solutions to their problems (Gregg, 1955). Reassured patients thus feel empowered to take control over their health. One study typified renewal of confidence with statements like “I was really worried before you told me that, but now I know that I have nothing to fear” (Teasdale, 1989). Reassurance instils hope in patients and offers patients optimistic viewpoints of any emotional or physical challenges they may be facing (Al‐Mutair et al., 2014; Fareed, 1996; Teasdale, 1989). Moreover, reassurance helps to keep intact the patient's pre‐illness identity, dignity and self‐esteem (Barr, 1992).

## 4 DISCUSSION

The review sought to synthesize existing studies to understand the application of the term “reassurance” as used in clinical practice. Reassurance emerged as a complex phenomenon characterized by three interconnected themes: (a) antecedents, (b) defining attributes and (c) outcomes. Emotional distress across the continuum of care was the main antecedent to the process of reassurance following which a connection between the nurse and the patient facilitated the resolution of the distress using verbal and non‐verbal approaches. The review findings highlight the concept of “reassurance” as an ongoing active process although it may appear latent to the nurse and patient. Our findings should increase awareness of what seems to be a “taken‐for‐granted” phenomenon and encourage nurses to reflect on and document their full reassurance episodes to facilitate better mapping of the process to emerging outcomes.

In medical practice, reassurance is often employed when discussing a patient's symptoms or diagnostic results (Kroenke, 2013; Redberg et al., 2011; Spence, 2018). The current review findings, however, suggest that the use of the concept of “reassurance” by nurses in clinical practice goes beyond these confines to include any episode of actual or potential emotional distress experienced by the patient and family, thereby highlighting a greater affinity of the concept to nursing practice. Additionally, the review findings suggest that reassurance transcends the physical state of the patient and family to connect emotionally with the nurse. These findings may be related to the core mandate of nursing which is to *care* instead of to *cure* (Watson, 1997). C*aring* endorses the professional identity of nursing, which provides avenues to respond to human dimensions of health and illness (Watson, 2007). Interestingly, how this important term (i.e. reassurance) is articulated in pre‐registration nursing education curricula and taught in nursing schools is rather vague. Although the concept may be resonated in specialist oncology, pain management, palliative and end‐of‐life care programmes (Buller et al., 2019; Linton et al., 2008; Wittenberg et al., 2018), the prevalence of emotional distress across patient groups makes it cogent to streamline the concept of “reassurance” in both undergraduate and graduate nursing curricula to prepare nurses. Perhaps, this may help to increase nurses’ self‐awareness and sensitivity to any clinical situation requiring reassuring interventions.

Further to the above, the review findings observed that nurses employed a variety of approaches to reassure patients and their families. These include verbal (such as words of encouragement and honest communication) and non‐verbal (therapeutic touch and active listening) approaches, which are similar to those employed by other practitioners (Giroldi et al., 2014; Pincus et al., 2013). The studies included in this review offer insight into the subjective usage of these approaches albeit how these were objectively carried out remain unclear. The authors agree that some of these approaches, particularly the non‐verbal approaches, represent the *art of nursing,* which may make it difficult to replicate or standardize. Aesthetic expressions such as empathy or therapeutic touches are often too complex to be reduced to a single definition that may make it difficult to express once the situation is over. However, as Carper asserts, the art of nursing or aesthetics remain one of the major patterns of knowing which cannot be ignored (Carper, 1978). Nurses should, therefore, be encouraged to reflect and document their reassurance experiences to strengthen the evidence‐base of these aesthetic expressions. By establishing a plethora of these experiences, common themes may be identified which relate to delivering reassurance. Of note, nursing is a process and interventions are mostly carried out by a team, hence the need to have a standardized way of reporting nuanced interventions such as reassurance (Johansen & Ervik, 2018; Kilner & Sheppard, 2010).

**FIGURE 2:**
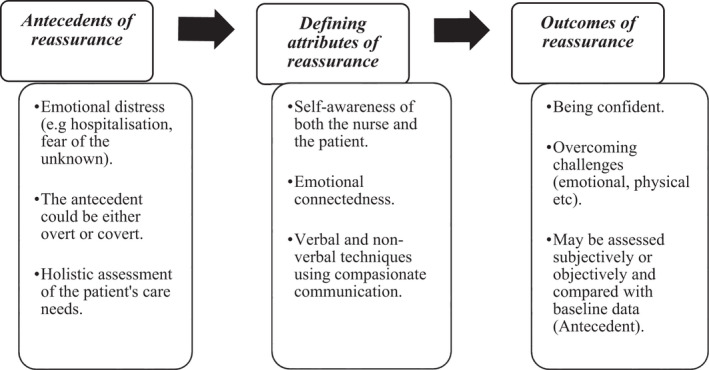
Conceptual framework of reassurance as understood from this review

Reassurance is a complex intervention that can lead to varied outcomes (Giroldi et al., 2014), including ensuring the dignity of the patients (Sailian et al., 2021). The more complex psychological and emotional care needs of a patient, the more the need for reassurance. Reassurance is at the heart of effective communication that ensures respectful and compassionate care as well as shared decision making in palliative and end‐of‐life care (Virdun et al., 2017). In the current review, patient empowerment and instilling hope emerged as outcomes associated with reassurance. Only one study used an objective measure to ascertain reassurance among persons with chest pains (Hicks et al., 2014). Outcome measures exist to evaluate anxiety, but as noted in this review, the concept of “reassurance” goes beyond anxiety which warrants the development of more situation‐specific outcome measures. Therefore, “outcome” in this context is part of the resource process rather than a stand‐alone step as illustrated in Figure 2.

### 4.1 A conceptual framework for reassurance in clinical care

Figure 2 illustrates the interconnectedness of the three themes that constitute the reassurance process. Nurses as health professionals should actively look out for signs that suggest that the patient is emotionally and psychologically unstable (*antecedents of reassurance*). Some patients may not verbalize what their worry is, while others will do. Therefore, the nurse should be approachable and be ready to listen to patients' concerns and actively look for both actual and potential sources of distress. This can be achieved by being self‐aware of the antecedents that warrant reassurance, by adopting empathetic connection with the patient through both expressed words and non‐verbal gestures (*defining attributes of reassurance*). The nurse should complete the reassurance process by evaluating the consequences of the act of reassurance (the *outcome of the reassurance*). If the evaluation indicates that the reassurance was not fully achieved, then the process is repeated. Therefore, reassurance is a *cyclical process* of problem identification, intervention and evaluation.

### 4.2 Strengths and limitations

This review presents a framework for reassurance in clinical care, and this could guide future research focusing on developing a tool for reassurance. The unique integration of evidence from reflective essays, theoretical papers and primary studies allowed us to present more nuanced and granular details on the use of reassurance in nursing practice. Moreover, the use of a highly sensitive search strategy, multiple electronic databases and no date filters reduced the likelihood of missing relevant papers.

However, an important limitation of the current review was the exclusion of non‐English papers, conference presentations and dissertations. This exclusion is likely to have resulted in the potential loss of some evidence.

### 4.3 Recommendations

Most reassurance interventions do not follow any evidence‐based framework, and therefore, there is a lack of consistency in reporting what was done and in evaluating the effectiveness of the nurse's reassuring actions. We recommend the development of an evaluation tool for reassurance. While we acknowledge that there are tools that currently assess the psychological state of distressed patients (anxious, stressed, depressed, among others), we believe that the perfunctory nature and use of reassurance requires a shorter validated assessment tool that could be rapidly used by nurses within the context of their busy work schedules. As suggested by Forbes (2009), documentation of nursing interventions should be characterized by the highest level of granularity. Future research should consider creating a checklist of items to report when documenting reassurance for both clinical and research purposes. Moreover, future studies should evaluate the various verbal and non‐verbal reassurance techniques in clinical trials to determine the best technique (or combination of techniques) and the factors that are likely to influence the success of specific reassurance techniques. This would help nurses adopt a more evidence‐based approach to reassurance.

## 5 CONCLUSION

This study reviewed the concept of reassurance as used in clinical practice and found three major themes: antecedents to, defining attributes of and outcomes of reassurance. Overall, this review reveals a stark lack of evidence about the standardization of the concept of reassurance for patients and their families in the clinical setting. Specifically, looking at what reassurance means from different perspectives (e.g. patients, family caregivers, healthcare professionals), settings (acute and long term facilities), type of disease (acute episode, chronic conditions and end of life), among others will harness the development of a standardized evidence‐based framework for reassurance that will be applicable in most context, and situations. This framework for reassurance could provide a guide for nursing education and practice, offering a flexible approach to the provision of compassionate and context‐appropriate reassurance to patients and families. Creating a checklist of items to report when documenting reassurance for both clinical and research purposes could be a consideration for future research.

## CONFLICT OF INTEREST

None declared.

## Supporting information

Supplementary Material

## Data Availability

As a systematic review, we relied solely on publicly published data. References of articles used in this evidence synthesis are provided throughout the paper. We have also provided our search terms to enable replication of our search strategy.
