# Supplementary material for: An integrative review of the use of the concept of reassurance in clinical practice
Source: Nurs Open. 2022 Mar 11;9(3):1515–35. doi: 10.1002/nop2.1102 (PMC8994970; doi:10.1002/nop2.1102)
Supplement: Supplementary file 1 — Supplementary Material [file NOP2-9-1515-s001.docx]

**Supplemental tables**

**Quality appraisal using MMAT**

**Qualitative studies**

|  | **Al-Mutair 2014** | **Karlsson 2012** | **Boyd 1989** | **Hermann et al., 2019** | **Jay, 1996** | **Jones et al., 2007** | **Gibb & O'Brien (1990).** | **Fareed 1996** | **Gustafsson 2018** | **Wocial et al 2014** | **Meyer et al 2019** | **Usher & Monkley 2001** | Brockwayet al., 1976 | **Chauhan, 2000** |
| --- | --- | --- | --- | --- | --- | --- | --- | --- | --- | --- | --- | --- | --- | --- |
| Are there clear research questions? | **Yes** | **Yes** | **Yes** | **Yes** | **Yes** | **Yes** | **Yes** | **Yes** | **Yes** | **Yes** | **Yes** | **Yes** | **Yes** | **Yes** |
| Do the collected data allow us to address the research questions? | **Yes** | **Yes** | **Yes** | **Yes** | **Can’t tell** | **Yes** | **Yes** | **Yes** | **Yes** | **Yes** | **Yes (partly)** | **Yes** | **Yes** | **Can’t tell** |
| Is the qualitative approach appropriate to answer the research question? | **Yes** | **Yes** | **Yes** | **Yes** | **Yes** | **Yes** | **Yes** | **Yes** | **Yes** | **Yes** | **Yes** | **Yes** | **Yes** | **Yes** |
| Are the qualitative data collection methods adequate to address the research question? | **Yes** | **Yes** | **Yes** | **Yes** | **Yes** | **Yes** | **Yes** | **Yes** | **Yes** | **Yes** | **Yes** | **Yes** | **Yes** | **Cant tell** |
| Are the findings adequately derived from the data? | **Yes (quotes were used)** | **Yes** | **Yes** | **Yes** | **Can’t tell** | **Yes** | **Yes** | **Yes** | **Yes** | **Yes** | **Yes** | **Yes** | **Yes** | **Yes** |
| Is the interpretation of results sufficiently substantiated by data? | **Yes** | **Yes** | **Yes** | **Yes** | **Can’t tell** | **Yes** | **Yes** | **Yes** | **Yes** | **Yes** | **Yes** | **Yes** | **Yes** | **Yes** |
| Is there coherence between qualitative data sources, collection, analysis and interpretation? | **Yes** | **Yes** | **Yes** | **yes** | **Can’t tell** | **Yes** | **Yes** | **Yes** | **Yes** | **Yes** | **Yes** | **Yes** | **Yes** | **Yes** |

**Quantitative descriptive**

|  | **Cossette et al (2002)** |
| --- | --- |
| S1. Are there clear research questions? | Yes |
| S2. Do the collected data allow to address the research questions? | Yes |
| 4.1. Is the sampling strategy relevant to address the research question? | Yes |
| 4.2. Is the sample representative of the target population? | Yes |
| 4.3. Are the measurements appropriate? | Yes |
| 4.4. Is the risk of nonresponse bias low? | No |
| 4.5. Is the statistical analysis appropriate to answer the research question? | Yes |

**RCT studies**

|  | **Hicks et al (2014)** |
| --- | --- |
| Are there clear research questions? | **Yes** |
| Do the collected data allow to address the research questions? | **Yes** |
| **Is randomization appropriately performed?** | **Yes** |
| Are the groups comparable at baseline? | **Yes** |
| **Are there complete outcome data?** | **Yes** |
| Are outcome assessors blinded to the intervention provided? | **Yes** |
| Did the participants adhere to the assigned intervention? | **Yes** |

**Mixed method studies**

|  | **Beaver 2005** | **Teaside & Kent, 1995** |
| --- | --- | --- |
| Are there clear research questions? | **Yes** | **Yes** |
| Do the collected data allow to address the research questions? | **Yes** | **Yes** |
| Is there an adequate rationale for using a mixed methods design to address the research question? | **No** | **Yes** |
| Are the different components of the study effectively integrated to answer the research question? | **Yes** | **Yes** |
| Are the outputs of the integration of qualitative and quantitative components adequately interpreted? | **Yes** | **Yes** |
| Are divergences and inconsistencies between quantitative and qualitative results adequately addressed? | **Can’t tell** | **Can’t tell** |
| Do the different components of the study adhere to the quality criteria of each tradition of the methods involved? | **Yes** | **Yes** |
